# Long noncoding RNA expression profile changes associated with dietary energy in the sheep testis during sexual maturation

**DOI:** 10.1038/s41598-017-05443-5

**Published:** 2017-07-12

**Authors:** Yanli Zhang, Hua Yang, Le Han, Fengzhe Li, Tingting Zhang, Jing Pang, Xu Feng, Caifang Ren, Shengyong Mao, Feng Wang

**Affiliations:** 1 Jiangsu Livestock Embryo Engineering Laboratory, Nanjing Agricultural University, Nanjing, 210095 China; 2 Jiangsu Key Laboratory of Gastrointestinal Nutrition and Animal Health, Nanjing Agricultural University, Nanjing, 210095 China

## Abstract

Spermatogenesis can be affected by nutrition, which operates through normal physiological processes by changing the testicular mass and hormone levels profoundly. However, little is known regarding how testis development is regulated by long noncoding RNA (lncRNA). In this study, we investigated the effects of high-grain (HG) feeding on testis development during sexual maturation mediated by lncRNA. The HG diet group showed an increase in growth hormone (GH), insulin-like growth factor-1 (IGF-1) and testosterone (T) levels, and in the number of sperm in the seminiferous tubules compared with the hay-fed group (*p* *<* 0.05). Moreover, we found 59 differentially expressed (DE) lncRNAs and 229 DE mRNAs in sheep testis between the two groups. qRT-PCR results of 20 randomly selected DE lncRNAs and mRNAs were also consistent with the RNA-seq data. Through functional enrichment analysis and lncRNA-mRNA interaction network analysis, we screened several lncRNAs that may be enriched for male reproduction such as spermatogenesis, sperm motility, steroid hormones, MAPK and ErbB signaling pathways. This study provides a first insight into the development of the testis with HG feeding in sheep and shows that these changes are associated with alterations in lncRNA expression.

## Introduction

In the current intensive sheep production system in China, most rams are fed high-energy diets after sexual maturation, in order to maximize their body weight gain. High-energy diets with adequate protein, vitamins, and minerals may also hasten testis development and spermatogenesis^1^. However, the process of spermatogenesis is complicated and involves strict developmental regulations at both the transcriptional and the post-transcriptional level^2^. It is difficult to have a thorough understanding of the effects of nutrition on spermatogenesis.

Among the regulators of spermatogenesis, recent years researchers have shifted their focus to post-transcriptional control mediated by noncoding RNAs (ncRNAs), which has emerged as an important regulator of spermatogenesis. The ncRNAs such as microRNA (miRNA)^3, 4^, PIWI-interacting RNA (piRNA)^5, 6^, small interfering RNA (siRNA)^7^ and lncRNA^8–10^ are known to play regulatory role in male germ cell development. Evidence revealed that the reductions in spermatozoal quality induced by under-nutrition were mediated by changes in expression of miRNAs and piRNAs^11^. A recent study also found miRNAs were involved in sheep abnormal reproductive morphology, apoptosis and male infertility response to under-nutrition^12^. Despite the findings, the understanding of how ncRNAs associated with effects of nutrition on spermatogenesis in the adult testis remains limited.

The lncRNAs are one of the most abundant ncRNA families which are more than 200 nucleotides in length. Studies have demonstrated lncRNAs as a new regulatory molecule are involved in mammalian development^13, 14^. Recently, many lncRNAs have been identified in specific developmental stages of testes and spermatogenesis in mouse, rat and human models. They are predicted to have critical roles in testicular development and spermatogenesis, such as *mrh1*, *Tsx*, *Dmr* and *HongrES2* ^15–18^. Therefore, we hypothesize that lncRNAs are also involved in the effects of nutrition on sheep testicular development. However, there is limited number of lncRNAs identified in sheep testes, which may be a bottleneck for further study of their complex physiological processes in sheep testicular development and spermatogenesis.

In order to gain insights into the relationships between lncRNA functions and testicular development responses to nutrition, we investigated the lncRNAs expression profiles of the testis from HG feeding and control hay feeding sheep. This is the first study to describe the expression profiles of lncRNAs in sheep testes. Our data will provide a useful resource for studying their functional roles in reproduction.

## Results

### Effect of high grain feeding on sheep body weight, hormone levels, testicular development and morphology

**Figure 1:**
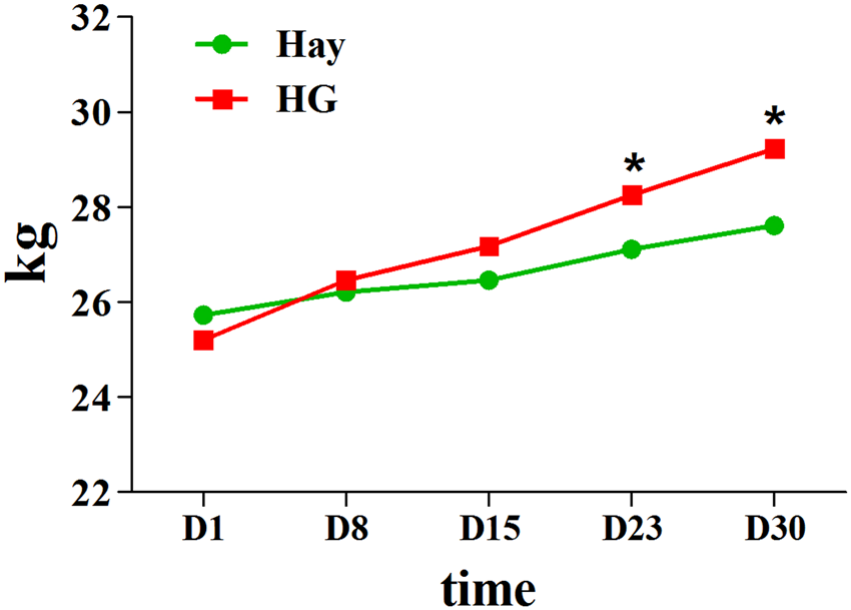
Weight change between hay diet and HG groups. The green and red lines represent the hay and HG groups, respectively; asterisks indicate significant differences between the groups (*p* < 0.05).

**Figure 2:**
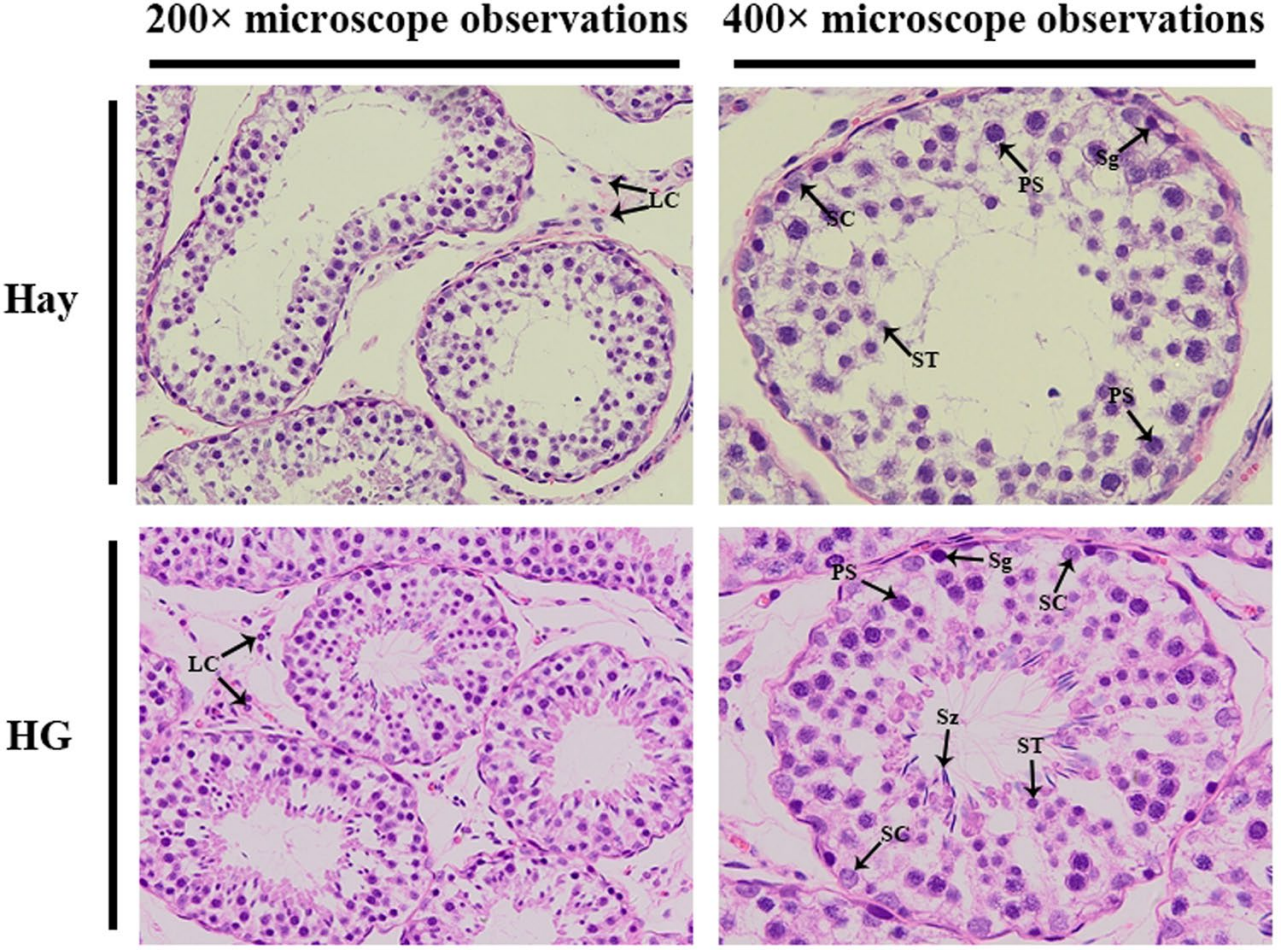
Histologic observations of sheep testes in the hay and HG diet groups. SC, Sertoli cell; LC, Leydig cell; Sg, spermatogonia; PS, primary spermatocyte; ST, spermatid; Sz, sperm.

**Figure 3:**
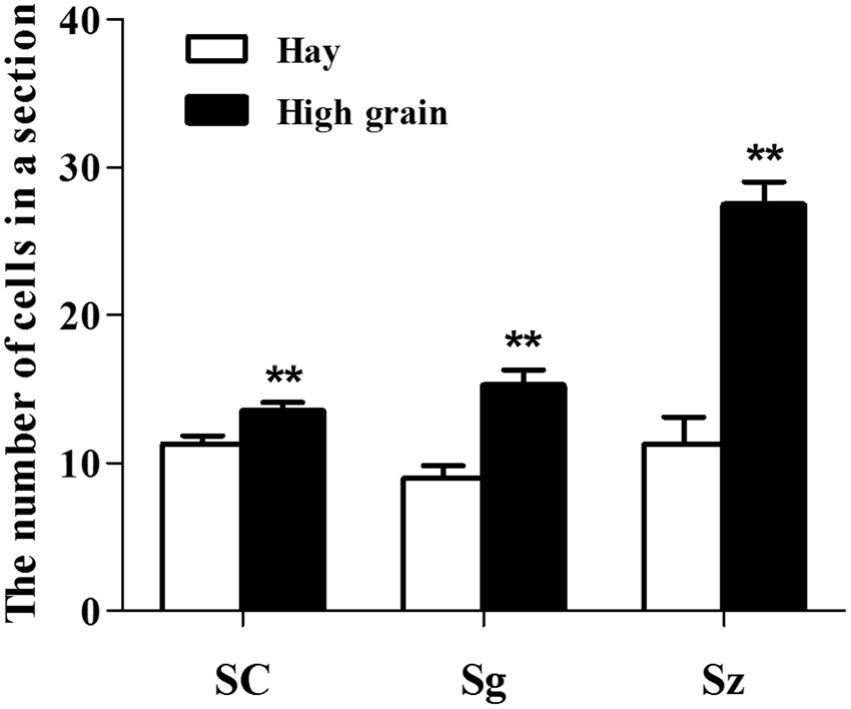
Analyses of the number of cells in seminiferous tubules. The number of Sertoli cells (SC), spermatogonia (Sg), and sperm (Sz) in the seminiferous tubules in the testes of sheep in the hay and high grain diet groups.

First, we examined the dietary effects on Hu sheep body weight, hormone levels, testicular growth, and germ cell number. Compared with the hay diet, the HG diet group had a significantly higher body weight (29.25 ± 0.95 kg *vs*. 27.61 ± 0.84 kg, *p* *<* 0.05) (Fig. 1). The HG diet group showed increased GH, IGF-1 and testosterone levels (*p* *<* 0.05, Table 1). Compared with hay diet group, testicle circumference and epididymis weight in HG diet group were significantly increased (13.08 ± 0.38 cm *vs*. 11.81 ± 0.40 cm, 29.83 ± 2.13 g *vs*. 23.62 ± 0.80 g, *p* *<* 0.05, Table 2). However, testis weight and testicle diameter were not significantly increased in the HG diet group compared with the hay diet group (*p* > 0.05). Furthermore, we observed histologic morphology in testicular tissues (Fig. 2), the number of sertoli cells (SC), spermatogonia (Sg) and sperm (Sz) in the seminiferous tubules are higher in the HG diet group than those in the hay diet group (*p* *<* 0.05, Fig. 3). These results suggested that HG feeding promoted the reproduction and growth-related hormone levels, it also increased sperm number in the seminiferous tubules. HG diet plays positive roles in sheep sexual maturity.

**Table 1:** Hormone levels of hay and HG groups.

Groups	T (ng/mL)			GH (ng/mL)			IGF-1 (ng/mL)		
	D3	D16	D30	D3	D16	D30	D3	D16	D30
Hay	0.77 ± 0.42	0.33 ± 0.07	0.39 ± 0.09^a^	0.92 ± 0.09	0.85 ± 0.05^a^	0.60 ± 0.08^a^	298.00 ± 7.12	236.71 ± 18.97	293.14 ± 24.92^a^
HG	0.84 ± 0.09	0.37 ± 0.07	2.09 ± 0.42^b^	1.01 ± 0.11	0.15 ± 0.12^b^	1.07 ± 0.11^b^	280.92 ± 7.09	275.92 ± 24.66	386.58 ± 29.08^b^

^a,b,c^Different letters denote statistically significant differences within each hormone (*p* < 0.05).

**Table 2:** Testicle indices of hay and HG groups.

Items		Groups		P-value
		Hay	HG	
Testis	Weight/g	122.58 ± 10.41	147.14 ± 12.46	0.161
	Percentage of live weight/%	0.44 ± 0.03	0.51 ± 0.05	0.291
	Circumference/cm	11.81 ± 0.40^b^	13.08 ± 0.38^a^	0.044
	Diameter/cm	6.44 ± 0.16	6.71 ± 0.23	0.363
Epididymis	Weight/g	23.62 ± 0.80^b^	29.83 ± 2.13^a^	0.021
	Percentage of live weight/%	0.09 ± 0.003	0.10 ± 0.01	0.121

^a,b,c^Different letters denote statistically significant differences within each index (*p* < 0.05).

### Overview of RNA sequencing in sheep testis

In order to identify the expression of lncRNAs in the sheep testes, two cDNA libraries for hay and HG were constructed and sequenced using the Illumina HiSeq 3000 Platform. A total of 101,897,574 and 97,180,382 raw reads were generated for the hay and HG diet groups, respectively. The GC contents of the libraries were 56.12% and 55.24%, respectively. The effective reads, with the rRNA removed from the clean reads, were mapped to ovis aries v4.0 reference genome. More than 79% of the reads were mapped to the genome. The number of sequences that had multiple positions mapped to the reference sequence was below 8%, but more than 75% of the reads were uniquely mapped to the reference sequence. Approximately 40% of the reads substantially mapped to the positive and negative chains of the genome (Supplementary Table S1).

Moreover, the number of reads that mapped to the exons (~70%) was significantly higher than the number that mapped to the intergenic and intronic region by annotated analysis (Supplementary Fig. S1). The reads mapped to the intronic region (7.91% to 11.42%) were most likely derived from the residues of pre-mRNA and introns during the alternative splicing process. Approximately one-fifth of the reads mapped to intergenic regions, which possibly due to incomplete gene annotation. These results suggested that the matching efficiency was high and most reads mapped to the exonic region.

### Identification of lncRNAs and mRNAs in sheep testis

**Figure 4:**
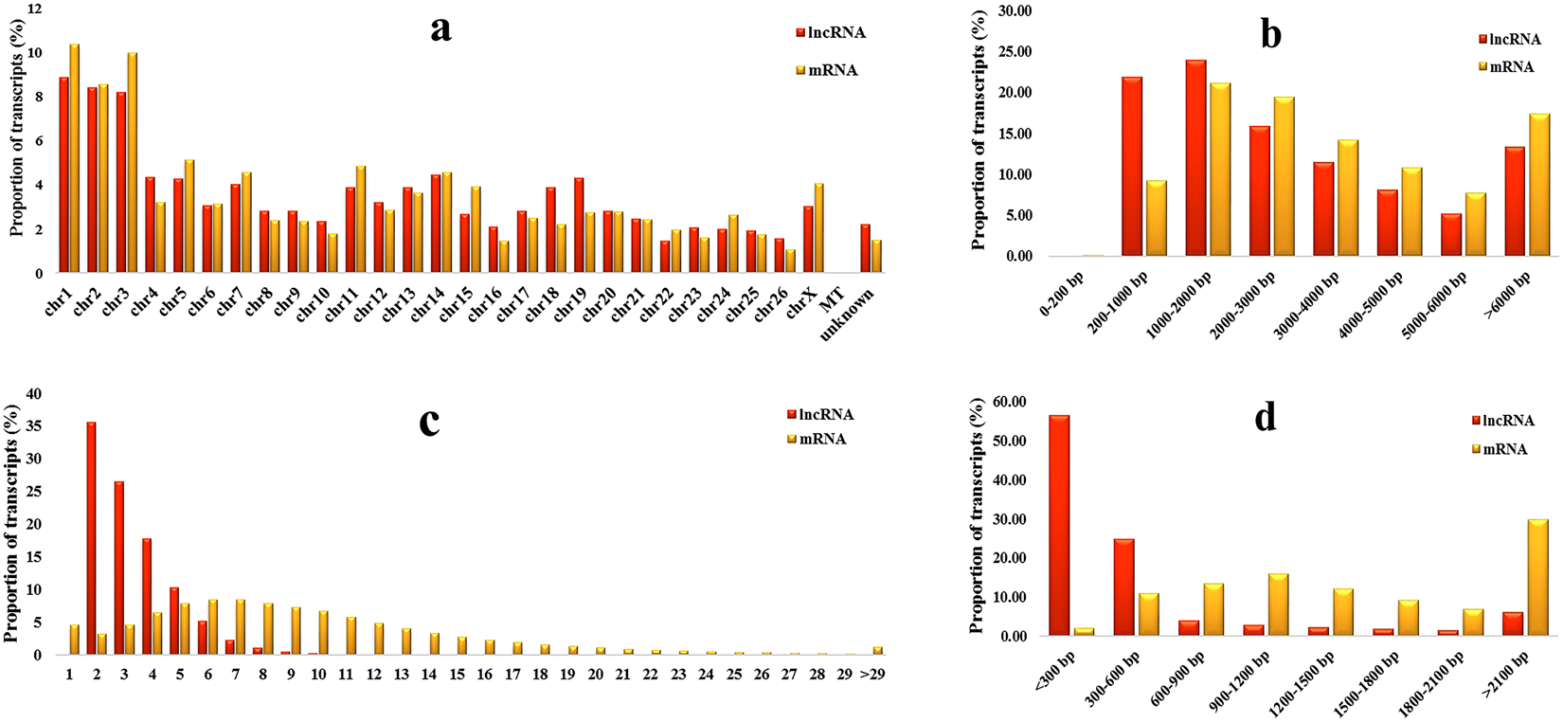
Identification of lncRNAs and mRNAs in sheep testes. (**a**) Distribution of lncRNAs and mRNAs in chromosomes. (**b**) Length of lncRNAs and mRNAs. (**c**) Exonic content of lncRNAs and mRNAs. (**d**) Length of the open reading frame of lncRNAs and mRNAs.

After mapping to the reference sequence, we identified 6,460 known lncRNAs and 42,300 mRNAs from 48,760 assembled transcripts. These transcripts were randomly distributed into 26 autosomes and the X-chromosome. We also found that one lncRNA was located in the mitochondria and that approximately 2.2% lncRNAs and 1.5% mRNAs were not matched to any chromosome location. The distribution patterns of the lncRNA and mRNA in sheep testis were similar, as shown in Fig. 4a. The lncRNA transcripts were mainly distributed from 200 bp–2000 bp, whereas the mRNA transcripts were mainly longer than 1,000 bp (Fig. 4b). In addition, the average number of exons of the lncRNA was less than that of the mRNA (3.40 *vs*. 9.55, respectively) (Fig. 4c). Furthermore, the average open reading frame (ORF) length of the lncRNA transcripts was 573 bp, within the range of 0 to 12,597 bp, which was shorter than that of the mRNA transcripts (Fig. 4d).

In addition, the lncRNA can be classified as lincRNA, antisense-lncRNA, and intronic-lncRNA types, whose proportions were 60%, 28% and 12%, respectively (Supplementary Fig. S2). These findings showed that the genomic information of lncRNA transcripts was different from that of the mRNA transcripts, except for the similar chromosome distribution.

### Profiling and verification of differentially expressed (DE) lncRNA and mRNA of the testis in the hay and HG diet groups

**Figure 5:**
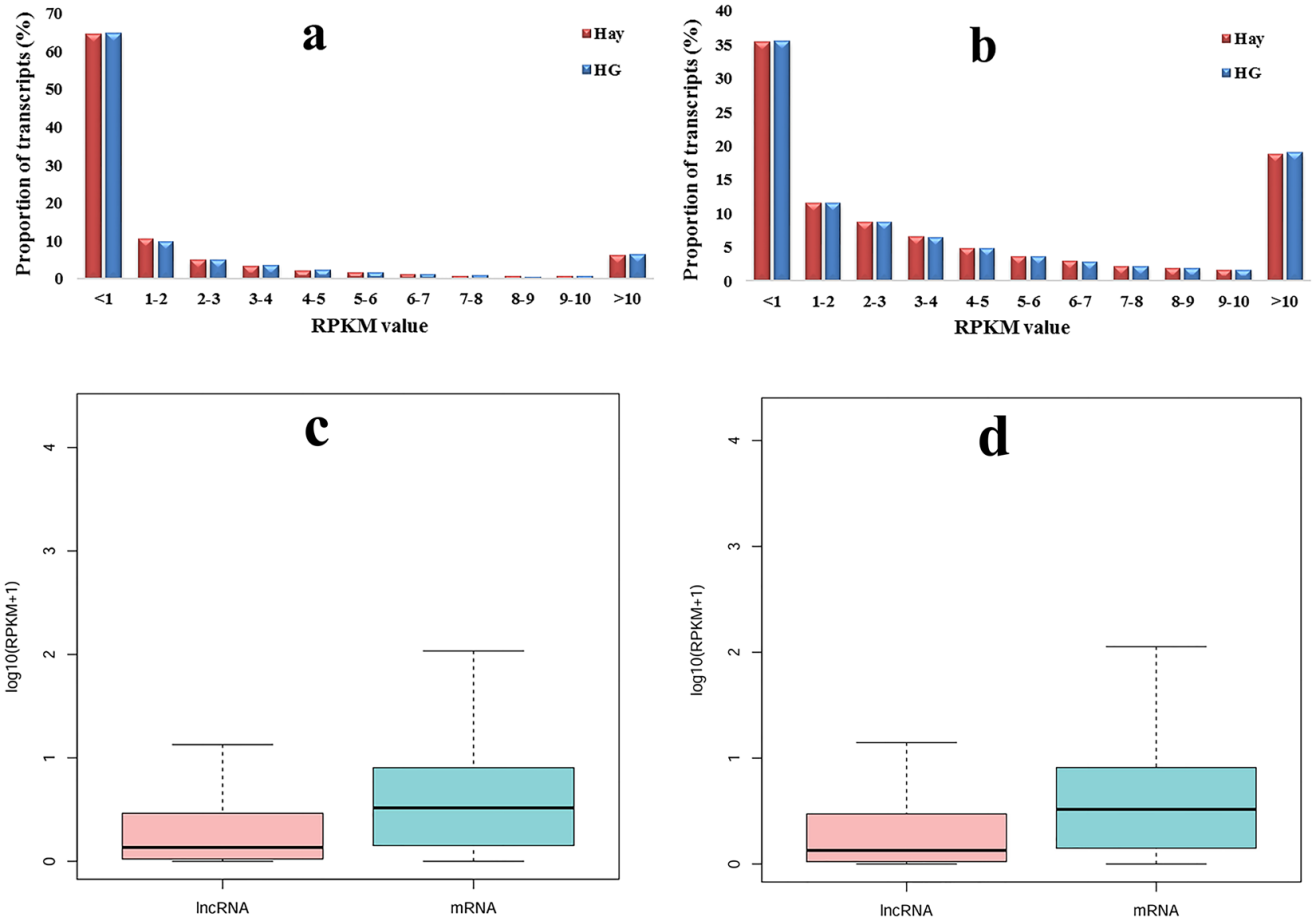
RPKM value of lncRNAs and mRNAs in sheep testis tissues. (**a** and **b**) represent the RPKM value of all the identified lncRNAs and mRNAs in the two groups, respectively. The boxplots in (**c** and **d**) show the expression levels of lncRNAs and mRNAs in the testis tissues of sheep in the hay and HG groups, respectively.

**Figure 6:**
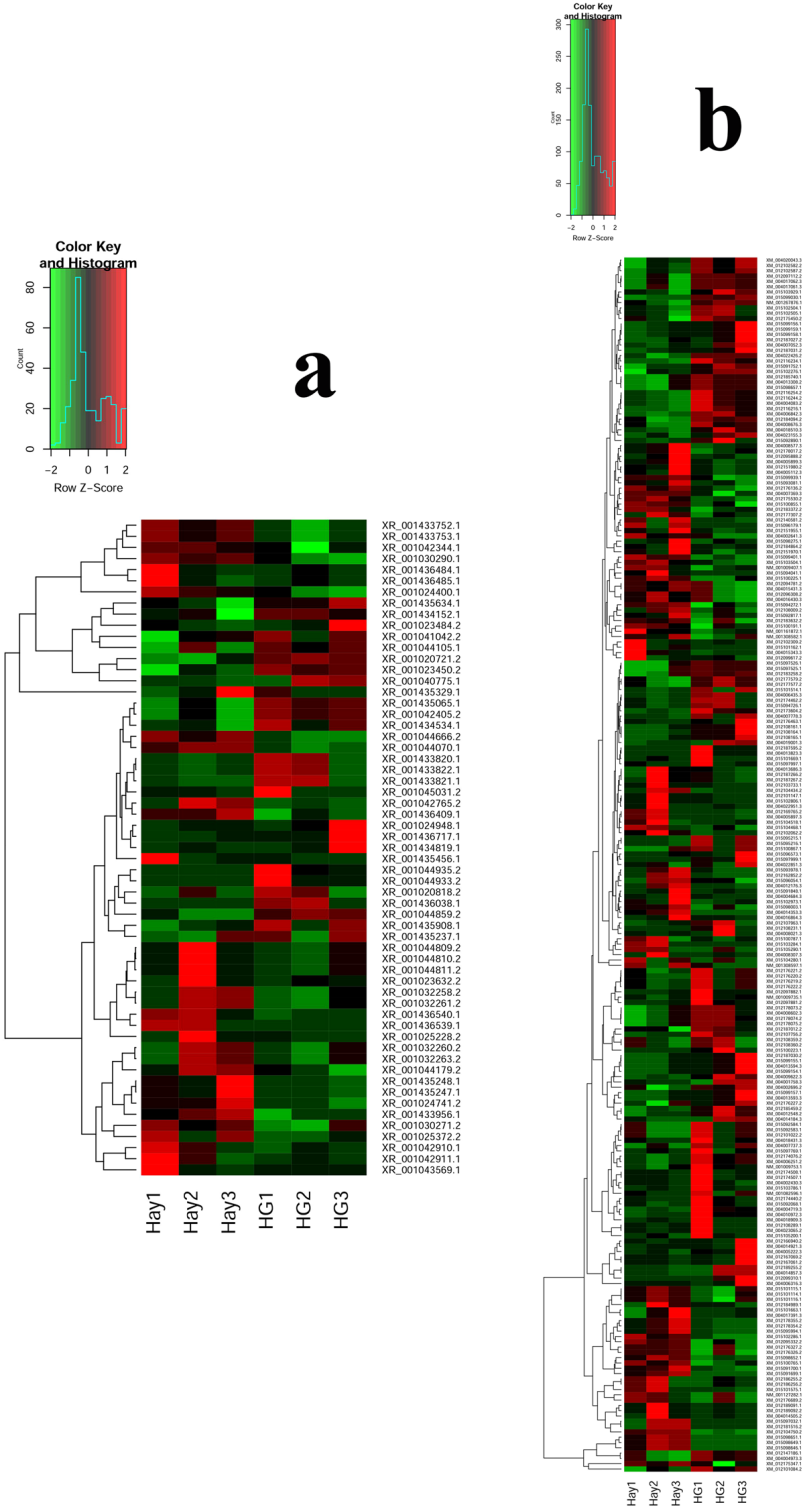
Heat maps illustrating the DE of lncRNA and mRNA between the hay and the HG diet groups. (**a** and **b**) show 59 DE lncRNAs and 229 DE mRNAs, respectively; green indicates downregulation and red indicates upregulation.

Next, the expression level of the lncRNA and mRNA transcripts was estimated using the RPKM value (Reads Per Kilobase per Million mapped reads). We found that approximately 65% of the lncRNAs and 35% of the mRNAs and transcripts expressed lower than 1 RPKM (Fig. 5a and b). The expression level of the lncRNA transcripts was relatively lower than those of the mRNA in both the hay and HG diet groups (Fig. 5c and d). In total, based on adjusted P-value threshold of <0.001 and |log2(fold change)| > 1, there were 28 upregulated and 31 downregulated lncRNA DE transcripts, and 127 up-regulated and 102 down-regulated mRNA DE transcripts in the HG group (Fig. 6a and b).

**Figure 7:**
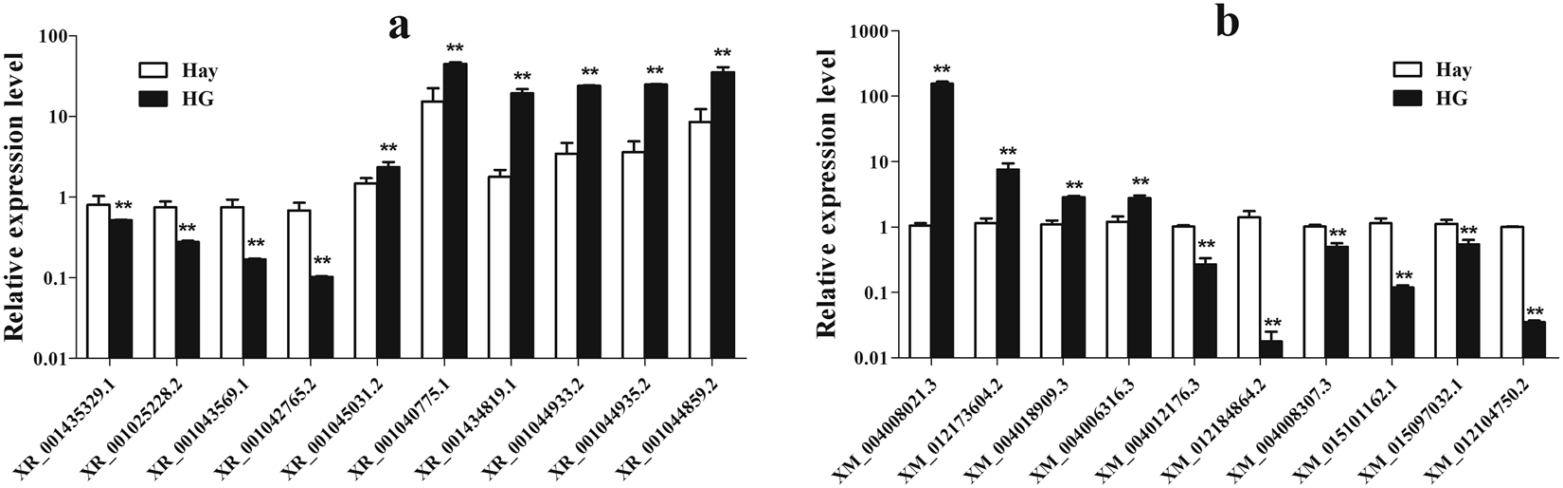
Verification of the expression level of ten DE transcripts in the hay and HG diet groups. The relative expression of DE lncRNAs and DE mRNAs in testis tissues was determined using qRT-PCR. (**a** and **b**) show the expression level of ten DE mRNAs and lncRNAs in the hay and HG diet groups, respectively. The experiment was biologically repeated 3 times and technically repeated 3 times for each group. The relative expression levels were normalized to the expression amount of GAPDH. The results are expressed relative to the hay group as the mean ± the SEM; *p < 0.05, and the Y-axis represents the values that were calculated by log10.

To further evaluate the reliability of RNA sequencing, 20 DE lncRNAs and mRNA transcripts were randomly selected to validate the relative expression levels in the sheep testis of the hay and HG diet groups using qRT-PCR. All these differential expression levels of lncRNA and mRNA were consistent with the RNA-seq results (Fig. 7a and b), which indicated that the RNA-seq data was reliable. These analyses also demonstrate that high-throughput sequencing has the advantage of detecting genes with low levels of expression (0 < RPKM < 1).

### GO and KEGG enrichment analysis of DE mRNAs annotated and DE lncRNAs target genes

**Figure 8:**
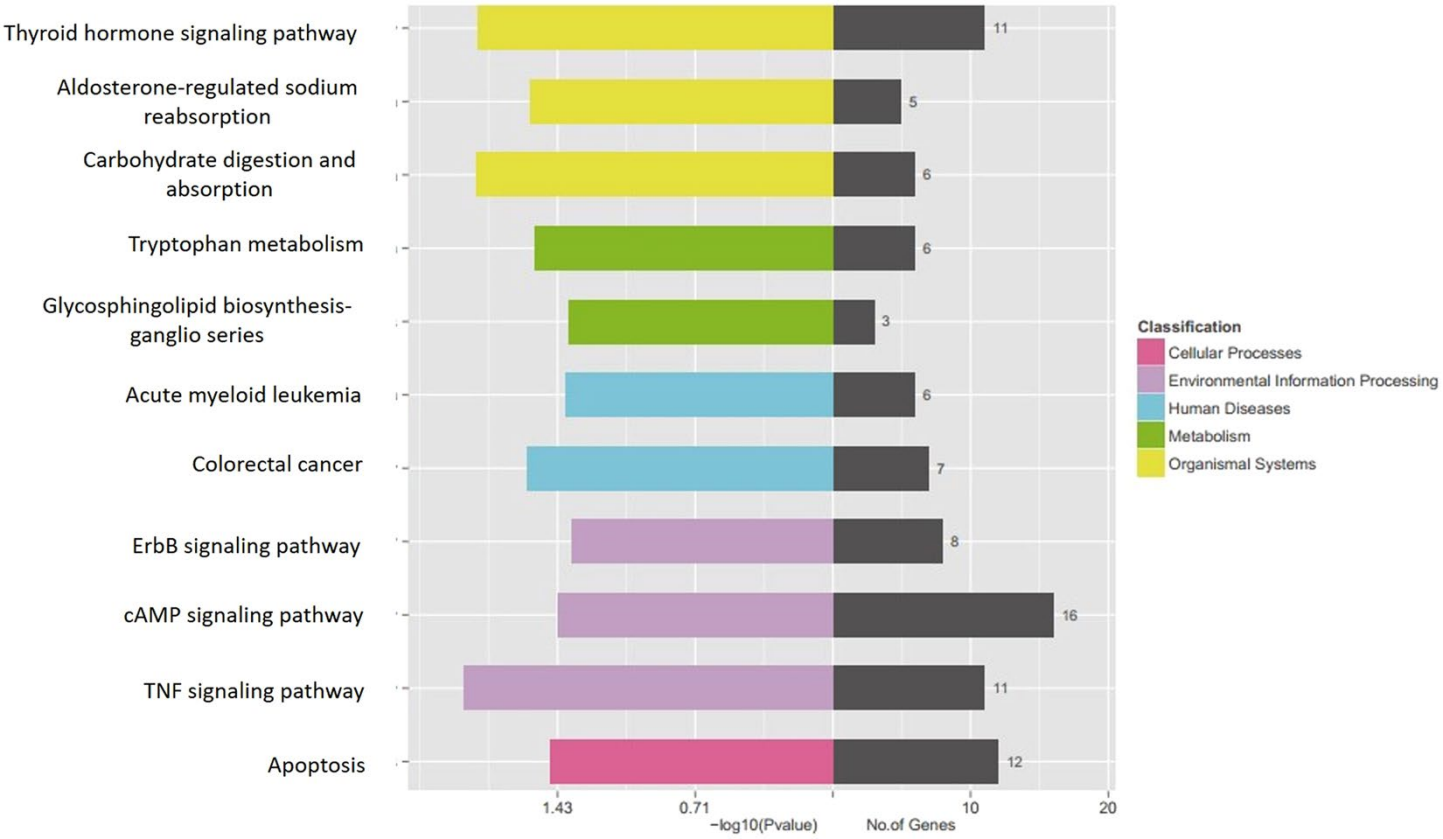
KEGG pathway analysis of target genes of DE lncRNAs. The KEGG pathway enrichment analysis was performed on the KEGG biological pathways database (http://www.genome.jp)^44–46^. The left side of the coordinate axis shows the P-values of the pathway, and the yellow, green, blue, purple, and pink colors indicate the KEGG classification: organismal systems, metabolism, human diseases, environmental information processing, and cellular processes. On the right side of the coordinate axis, the gray color represents the number of genes enriched in the pathway.

93 upregulated and 93 downregulated DE mRNAs that have been annotated were screened for GO enrichment analysis. The results showed that the most enriched GOs were involved in 405 biological processes, 94 cellular components, and 155 molecular functions (Supplementary Fig. S3). The KEGG pathway analysis indicated that some DE genes, such as *BMP2*, *RASGRF2*, *TSHZ2* and *CREB3L3* were involved in male reproduction-related pathways. As shown in Fig. 8, signaling pathways such as thyroid hormone, ErbB, cAMP and apoptosis were among the ten most significantly differential KEGG pathways of the DE lncRNAs target genes. These data indicated that the related lncRNAs might be potentially involved in sheep testicular development.

### Co-expression analysis of DE lncRNA-mRNA

To preferentially filter lncRNAs that regulate male reproduction, 59 lncRNA transcripts and 44 mRNA transcripts that were enriched in male reproduction-related pathways were used for the co-expression network analysis by the cytoscape software. We found that 43 DE lncRNAs were associated with 21 male reproduction-related mRNA. There are three co-expression relationships: mRNA-mRNA, mRNA-lncRNA, and lncRNA-lncRNA (Supplementary Fig. S4). The reproduction-related targeted genes and their co-expressed lncRNAs are partially listed in Table 3. The network pattern suggested that each gene was co-expressed with multiple lncRNAs, indicating the inter-regulation of lncRNAs and mRNAs in testicular development.

**Table 3:** Reproduction-related genes and their co-expressed lncRNAs.

Gene symbol	Up-/Downregulated	Chromosomal location	Co-expressed lncRNAs	Up-/Downregulated	Chromosomal location	COR	P-value
LOC101105256	Down	chr7	LOC105609981	Down	chr2	0.96012	0.01111
			LOC105610224	Down	chr12	0.87617	0.03056
			LOC105613108	Down	chr9	0.98507	0.03194
			LOC105616588	Down	chr12	0.95989	0.025
SLC22A20	Down	chr21	LOC105607399	Down	chr10	0.96239	0.03472
			LOC105610178	Up	chr2	−0.9241	0.00694
GABRP	Down	chr16	LOC106991431	Down	chr11	0.99492	0.0375
ELSPBP1	Down	chr14	LOC105604187	Down	chr21	0.96198	0.01667
			LOC105610322	Down	chr9	0.97661	0.00556
DIRAS3	Down	chr1	LOC106991389	Up	chr10	−0.9464	0.00833
			LOC106991859	Up	chr22	0.85935	0.03333
			LOC106991860	Down	chr22	0.85181	0.01389
PAPPA2	Up	chr12	LOC105605113	Down	chr26	0.95722	0.00556
			LOC105607399	Down	chr10	0.85183	0.03889
			LOC105608706	Down	chr2	0.96997	0.00417
			LOC105609364	Up	chr14	0.9984	0.00972
			LOC105613844	Up	chr14	−0.8841	0.00556
KRT18	Up	chr3	LOC105605281	Up	chr26	0.99171	0.02778
			LOC106991234	Up	chr6	0.99897	0.04167
ABCD2	Up	chr3	LOC105608131	Up	chr14	0.99690891	0.05
			LOC105609364	Up	chr14	0.99598424	0.0375
CCR6	Up	chr8	LOC105602173	Up	chr15	0.9117	0.00139
			LOC105604811	Down	chr24	−0.8767	0.02083
			LOC105610322	Down	chr9	−0.8958	0.02083
			LOC106991860	Down	chr22	−0.9057	0.03889
RASGRF2	Down	chr5	LOC101120392	Down	unknown	0.9870	0.0134

### Roles of screened DE lncRNAs in the development of the sheep testis

**Figure 9:**
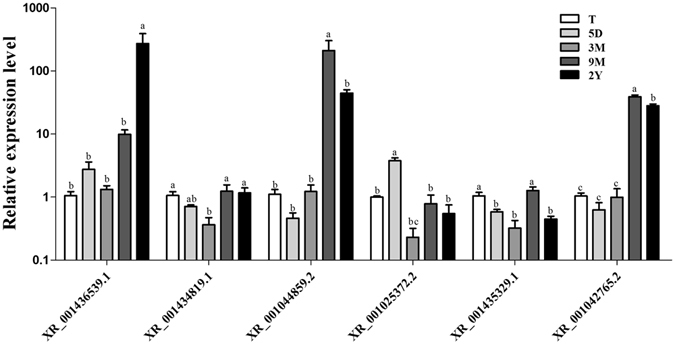
The expression level of six randomly selected differential expressed lncRNAs of different ages. The relative expression of screened DE lncRNAs in testis was determined using qRT-PCR. T, 5D, 3 M, 9 M, and 2Y represent prenatal embryo at 110 days and postnatal at 5 days, 3 months, 9 months, and 2 years. The experiment was biologically repeated 3 times and technically repeated 3 times for each group. The relative expression levels were normalized to the expression amount of *GAPDH*. Results are expressed relative to the prenatal embryo at 110 days as the mean ± the SEM. (**a**,**b**,**c**) Different letters denote statistically significant differences within each group; *p < 0.05, and the Y-axis represents the values that were calculated by log10.

Next, to further validate whether these DE lncRNAs play roles in male reproduction, we selected six DE lncRNAs whose targeted genes were *DIRAS3*, *KRT18*, *PAPPA2*, *RASGRF2*, *GABRP*, and *LOC101105256*. They were all enriched for male reproduction-related pathways. Then the expression levels of the six selected lncRNAs in testis tissues of sheep of pre- and postnatal ages were examined (Fig. 9). One of the observation is that the expression level of *XR_001436539*.*1* increased in sheep testis tissues with age. Interestingly, we also found that these six DE lncRNAs in testis tissues showed significantly different expression patterns with age (*p* < 0.05), which suggested that these lncRNAs might play roles in testis development.

## Discussion

LncRNAs have gained widespread attention as a novel regulatory player in cellular development over the past few years owing to high-throughput transcriptome analysis^19^. lncRNAs are abundant in testis, while the overall number undergoes developmental changes are similar to mRNAs^10, 20, 21^. Some studies found that lncRNAs played important roles in mammalian testis development and spermatogenesis. Numerous lncRNAs from testis tissue or germ cells of mouse^22^, porcine^23^, and human^24, 25^ have been identified. To date, however, there are few study on the lncRNA in sheep testis, especially Hu Sheep. Hu Sheep is a Chinese endemic species with high prolificacy and good adaptability to a hot and humid climate. They can be raised indoors all year round. In this study, we not only provided the first profile of lncRNA in sheep testis but also investigated the possible roles of lncRNA in testis development that respond to high-grain feeding.

The effects of nutrition on testis mass in male farm animals have long been known, as it has the direct relationship between testicular mass and sperm production^12^. In previous studies, evidence indicated that nutrition can influence male reproduction. For example, Holstein bulls fed high-energy diet showed advanced testicular maturation^26^. In mature male sheep and goats, changes in feed intake can induce changes in the size of the seminiferous tubules and spermatogenic efficiency^1^. Underfeeding in male sheep increased the number of apoptotic germ cells and the expression of apoptosis-related genes in testicular tissue^11^. Nutrition at different levels can affect the function of Sertoli cells, which mediate spermatogenesis in sexually mature sheep^27^. Our results also showed that there were some differences between the hay and HG diet groups after one month of feeding. The responses to nutrition include increased testosterone levels, testicle circumference and number of SC, Sg, and Sz. Our results suggested that short-term high-grain feeding had a positive role in male reproduction during sexual maturation.

Although many potential lncRNAs have been identified in male germ cell development, only a few have been functionally annotated with male reproduction. They are key for spermatogenesis and testis development. For example, lncRNA *mrhl* can negatively regulate the Wnt signaling pathway, which is important in regulating mouse spermatogenesis by binding to chromatin and affecting the expression of meiotic differentiation marker genes^28^. However, we didn’t find *mrh1* orthologue in sheep testis by chromosome synteny. In this study, we found the similar distribution of chromosome locations of mRNA and lncRNA indicated that interactive lncRNAs were close to mRNAs. The average length of lncRNAs and mRNAs in sheep testis were longer than those in porcine (1,713 bp and 1,983 bp, respectively)^23^. The expression level of lncRNA was lower than that of mRNA, which was consistent with porcine testes. In addition, approximately 35% of the lncRNAs were below a 1 FPKM value.

A total of 229 mRNAs and 59 known lncRNAs were significantly differentially expressed between the hay and HG diet groups. In addition, we identified 9,866 new lncRNAs and 68 new DE lncRNAs in the two groups, including 41 upregulated and 27 downregulated lncRNAs. To further validate the RNA-Seq results, qRT-PCR was performed to evaluate the expression patterns of 20 known lncRNAs and mRNAs. The results were consistent with those of the RNA-seq. Furthermore, by referring to the published RNA-Seq data from seven cell types during mouse spermatogenesis^29^, we identified 6 lncRNAs and their 4 target DE genes *ILR5A*, *OAS2*, *PROZ*, and *TSHZ2*. They expressed in type A spermatogonia, type B spermatogonia, pachytene spermatocyte, round spermatids, and elongated spermatids (Supplementary Table S2). This finding suggests that these lncRNAs may be important in sheep spermatogenesis.

GO is a bioinformatics initiative that is widely used to study functional relationships between genes. In our study, GO analysis were performed using all differentially expressed mRNAs. The results showed that 44 mRNA transcripts were enriched for three GO categories: biological process, molecular function and cellular component. In the biological process category, the upregulated genes, such as *SERPINF2*, *ESRRG*, *LAX1* and *LOC105611720* were involved in some GO terms like spermatogenesis, sperm motility, inactivation of MAPK activity and steroid hormone-mediated signaling pathway, positive regulation of cell differentiation, ERK1 and ERK2 cascade. The downregulated genes, such as *LOC101123097*, *BMP2*, *RASGRF2*, *ELSPBP1*, *DIRAS3* and *LOC101112345* were involved in the cholesterol biosynthetic process, cholesterol efflux, cholesterol homeostasis, cholesterol import, response to estrogen, and reverse cholesterol transport. In the molecular function category, the downregulated genes *LOC101105256*, *TRHR* and *LOC101112345* were associated with the androgen receptor binding term, thyrotropin-releasing hormone receptor activity term and cholesterol binding and cholesterol transporter activity terms. The upregulated gene *ESRRG* was also involved in steroid binding and steroid hormone receptor activity terms, which were enriched for the synthesis of reproductive hormone.

Furthermore, KEGG analysis confirmed that some DE genes were associated with several reproduction-related signaling pathways, such as Hedgehog, TGF-β, Hippo, estrogen hormone synthesis, TNF, AMPK, and PI3K-Akt, which were similar to the results of transcriptome analysis of testes from boars^30^. However, how these genes play roles in male reproduction remains largely unknown. To further understand the functions of lncRNAs, we applied a pathway analysis of target genes to study the DE lncRNAs and found that TNF, Thyroid hormone, Apoptosis, cAMP, and ErbB signaling pathways have been reported to be associated with male reproduction^31–34^.

To further understand the lncRNAs biological functions in sheep testis, we selected those DE lncRNA target genes that have been reported to be associated with male reproduction, such as the TNF, Thyroid hormone, Apoptosis, cAMP, and ErbB signaling pathways, to construct a co-expressed network. Within this co-expression network, the positive and negative pairs of lncRNAs and mRNAs suggested that these lncRNAs may regulate testis development because of its co-expressed targeted mRNAs affecting male reproduction. For example, *SLC22A20* has been demonstrated to have important roles in the maintenance of testicular steroidogenesis^35^, which indicated that the co-expressed lncRNA *LOC105607399* and *LOC105610178* likely have these functions. However, further research is needed. In addition, *DIRAS3* was enriched for genetic imprinting terms, and lncRNA *GNG12*-*AS1* has been verified as being co-expressed with *DIRAS3* in several tissues and coordinately downregulated with *DIRAS3* in breast cancers^36^. For example, *LOC101105256*, which is a significantly downregulated gene in the HG diet group, was enriched for androgen receptor binding, transcription coactivator activity, and the androgen receptor signaling pathway. This observation will be meaningful for exploring the function of co-expressed lncRNAs.

Furthermore, we verified the presence of DE lncRNAs and mRNAs in sheep testis tissues. By examining the patterns of DE mRNAs with known functions in male reproduction, such as upregulated genes *CCR6*, *ABCD2*, *PAPPA2*, and *KRT18* in the HG diet group^37–40^, we speculated that those lncRNAs co-expressed with these genes in our study might contribute to testicular response to nutrition by regulating these coding genes.

In summary, we demonstrated the expression profiles of mRNAs and lncRNAs in sheep testis in order to further our understanding of their regulatory roles in sheep testes development and spermatogenesis. We identified a collection of aberrantly expressed lncRNAs in sheep testis tissues that were fed a HG diet compared with a hay diet. Our study might contribute to find out mechanism that how nutrition effects spermatogenesis in sexually mature sheep. In addition, further study of these lncRNAs could provide useful insights into sheep testis development.

## Methods

The experimental design and procedures were performed in accordance with the approved Guidelines for Animal Experiments of Nanjing Agricultural University, China and were approved by the Animal Care and Use Committee of Nanjing Agricultural University, China (Approval ID: SYXK2011-0036).

### Animals, experimental design, and sample collection

Twelve Hu male sheep aged 6 months with similar body conditions were used in this study. The sheep were pre-fed a hay diet with alfalfa and oats for 28 days to ensure adaptation to the low-energy hay diet prior to the experiment. Then, the sheep were randomly divided into two groups and fed either a hay diet (alfalfa and oats, 0% grain; n = 6) or HG diet (60% grain; n = 6). They were placed in individual pens with free access to water for one month. Adaptation to the HG diet was carried out with the proportion of grain in the feed gradually increasing over the 4 days to 60%. The diet formula is shown in Supplementary Table 3, and the feeding scheme used for the experiment is shown in Supplementary Fig. 5.

On day 60, the sheep were euthanized, and blood samples were collected via jugular vein and the testes were immediately removed (within 5 minutes). Then the weight, circumference, and diameter of the testes were measured. A quarter of the left testis near the epididymal tail from each sheep was collected with RNAlater and snap frozen in liquid nitrogen immediately for RNA extraction.

### HE staining

The testis tissues were washed with 0.9% saline and were fixed with 4% paraformaldehyde for 48 h at room temperature and then embedded for further histologic analysis. Next, 5-µm-thick sections were stained with hematoxylin-eosin, and the morphology of the testis was analyzed under a microscope. The cells number of SC, Sg and Sz were counted at 200× magnification from 30 randomly selected seminiferous tubule cross-sections from each group as previously described^30^.

### Hormone assay

The blood was centrifuged at 3,000 rpm for 10 minutes, and then the supernatant was collected and stored at −20 °C. GH, IGF-1 and T levels were analyzed by radioimmunoassay (RIA) following the instructions of an iodine [125I] human growth hormone radioimmunoassay kit, an insulin-like growth factor I detection reagent, and an iodine [125I] testosterone radioimmunoassay kit (Beijing North Biotechnology Research Institute, China), respectively.

### RNA extraction and Real-time quantitative PCR

The total RNA of each testis tissue was isolated with Trizol reagent (Invitrogen Life Technologies, Carlsbad, California, USA). The purity (optical density value, OD value) and concentration of 1.5 µL RNA was determined using NanoDrop instrument (NanoDrop Technologies, Wilmington, DE, USA). The RNA products were also analyzed using 2% agarose gel electrophoresis, stained with ethidium bromide and visualized under UV illumination with 50ms exposure time (see Supplementary Fig. S6). The cDNA was then synthesized using a reverse transcription (RT) reagent kit with gDNA Eraser (Takara, Dalian, China). qRT-PCR was performed to detect the expression of genes on a StepOnePlus Real-Time PCR System (Life Technologies, USA) using SYBR Green Master mix (Roche Applied Science, Mannheim, Germany) in a reaction volume of 20 µL: SYBR 10 µL, RNase-free water 7.8 µL, cDNA 1 µL, forward primer 0.6 µL, and reverse primer 0.6 µL. The steps included a holding stage at 50 °C for 2 min, 95 °C for 10 min, then 35 cycles of 95 °C for 15 sec, 60 °C for 30 sec, and 72 °C for 30 sec, followed by 95 °C for 15 s, 60 °C for 1 min, and 95 °C for 15 s.

First, expression level of the housekeeping gene actin-beta (*ACTB*) was normalized to glyceraldehyde 3-phosphate dehydrogenase (*GAPDH*). And expression level of *GAPDH* was normalized to *ACTB*. Results showed that GAPDH expression was similar in HG and hay groups (see Supplementary Fig. S7). Then, the comparative quantification of each RNA was normalized to *GAPDH* using the 2^−ΔΔCt^ method for further analysis. Housekeeping gene and ten differentially expressed lncRNAs and mRNAs primers are shown in Table 4, respectively. The specificities of the primers were confirmed by PCR amplification and agarose gel electrophoresis (see Supplementary Fig. S8).

**Table 4:** The primer of GAPDH, ACTB, DE lncRNAs and mRNAs.

	Gene symbol	Primer Sequence	Product size (bp)
Primers of GAPDH and ACTB	ACTB	F:TCAGCAAGCAGGAGTACGAC	
		R:ACGAGGCCAATCTCATCTCG	137
	GAPDH	F:GTCAAGGCAGAGAACGGGAA	
		R:GGTTCACGCCCATCACAAAC	232
Primers of ten DE lncRNA	XR_001045031.2	F:CTCCCAAAGAAGCCTGAATG	
		R:CCCAATAGAGCAATCCCATC	120
	XR_001040775.1	F:ACTACCTGGAACACCTGAAACC	
		R:TTGGACACGACTGAAGCAAC	138
	XR_001434819.1	F:GCAAGCACACCAAAAATCAC	
		R:AACCTGCCCCTCTTACCTGT	130
	XR_001044933.2	F:AGAGGCATTTTCAGACGGGG	
		R:GGCCCATCACCACAATCGTA	116
	XR_001044935.2	F:AGAGGCATTTTCAGACGGGG	
		R:GGCCCATCACCACAATCGTA	116
	XR_001044859.2	F:TTTGGGACCTTGACATCCTC	
		R:GTTGGGAACAGAGTGCCTTG	122
	XR_001435329.1	F:CTCACCCAAGAGGGAACCTG	
		R:TGACAAACTGAGCCCCTTCC	251
	XR_001025228.2	F:TCGCCTAATGGTTCTTCGTC	
		R:GATGTGCTTTGTGGATGTGG	114
	XR_001043569.1	F:TGCAGTCTTCGTGCTCACAT	
		R:GGCCAGTCAGCTTCATCCTT	199
	XR_001042765.2	F:GATGAGAGCACACAGCGTCT	
		R:CTCCCCTACTGGAAAACGCA	116
Primers of ten DE mRNA	XM_004008021.3	F:GCGTTCCTGTGGGATTTTT	
		R:TGCTTCAGATGTCGGGATAA	116
	XM_012173604.2	F:AGGCAGTTTAGACAACGCCA	
		R:ACTGCTTGCTGTTTGGGTCT	130
	XM_004018909.3	F:CAAACACTGGGAGCCTGAGA	
		R:GCCTTGGATGATGAAGATGG	124
	XM_004006316.3	F:CAGGTCAAAGACTGGGGACA	
		R:AGCAGCAAGACGGGCATT	120
	XM_004012176.3	F:TGGGAGGAATGGATAACAACA	
		R:TAACAGCCACACCAAACTGC	120
	XM_012184864.2	F:TCGTGAGAAATTTGCCCAGT	
		R:GCAGCAACTCATATGGCTCA	131
	XM_004008307.3	F:AAGTCGCCAACTTCGTGGAT	
		R:GGTTGTGGACGTCGTACTGT	159
	XM_015101162.1	F:TATCGACAGCTTCTGTGATGTC	
		R:CGGCTTCTCAGGTTCAGTAGG	162
	XM_015097032.1	F:TGTGCAGACTGACCTACCAC	
		R:CACAACATGGGCAGCTCTTTG	207
	XM_012104750.2	F:CAGCACATCCCAACATCG	
		R:CAGCACATCCCAACATCG	117

### Library construction and sequencing

Total RNA was extracted from three sheep testicular tissue in each group, and ribosomal RNA was removed using the Ribo-Zero™ kit (Epicentre, Madison, WI, USA). Fragmented RNA (the average length was approximately 200 bp) and cDNA were then synthesized and purified by reverse transcription. After PCR amplification and purification using the Qubit® dsDNA HS Assay Kit, fragment length of approximately 260 bp was chosen for library construction using the NEBNext® Ultra™ RNA Library Prep Kit. The libraries were paired-end sequenced (PE150, Sequencing reads were 150 bp) at the Ribo Bioinformatics company (Guangzhou, China) using Illumina HiSeq 3000 platform.

### Identification of lncRNA and mRNA

The raw data were first filtered to remove low-quality reads, then the clean data that passed repeated testing was assembled using the Cufflinks and Scripture based on the reads mapped to the reference genome (Ovis aries v4.0). The assembled transcripts were annotated using Cuffcompare program from the Cufflinks package. The unknown transcripts were used to screen for putative lncRNAs. Three computational approaches, CPC/CNCI/Pfam^41, 42^, were combined to sort nonprotein-coding RNA candidates from putative protein-coding RNAs in the unknown transcripts. Putative protein-coding RNAs were filtered out using a minimum length and exon number threshold. Transcripts with lengths above 200 nt with more than two exons were selected as lncRNA candidates. They were subjected to further screening using CPC/CNCI/Pfam to distinguish the protein-coding genes from the noncoding genes. In addition, the different types of lncRNAs, including lincRNA, intronic-lncRNA, and antisense-lncRNA, were selected using cuffcompare.

### Differential expression analysis

The RPKM value was used to estimate the expression levels of genes. The number of DE genes detected between the two groups using Gflod software^43^, and the counts and RPKM values were also calculated. Then, the statistically significant DE genes were obtained by an adjusted P-value threshold of <0.001 and |log2(fold change)| > 1 using the Audics software. Finally, a hierarchical clustering analysis was performed using the R language package gplots according to the RPKM values of differential genes in different group. And colors represent different clustering information, such as the similar expression pattern in the same group, including similar functions or participating in the same biological process.

### GO terms and KEGG pathway enrichment analysis

Based on the KEGG biological pathways database (http://www.genome.jp) and the P values that were calculated by Fisher’s Exact Test and 0.05 being defined as the significant threshold, the genes were screened and enriched for the pathways. Next, the significance of the pathway enrichment analysis was corrected by FDR, and the corrected P-value (Q-value) was obtained. Following KEGG pathway enrichment analysis, both genes and pathways were screened. To further study the regulative function of lncRNAs, their target genes were predicted by the NCBI, Gencode, Rfam, and lncRNAdb databases. According to the pathway enrichment analysis of the target genes, lncRNAs could be involved in the candidate pathways. The GO annotation information was analyzed using the Gene Ontology database^47^, which was similar to the KEGG pathway analysis. The functions of DE genes were investigated by the significance analysis of a gene set on GO categories.

### Co-expression network construction and prediction of target genes of DE lncRNAs

A co-expression network of DE mRNAs and lncRNAs was constructed by calculating the Pearson correlation coefficient and P value between multiple genes. In our study, the transcripts were filtered using a COR of >0.85 and a P-value of <0.05. Then we chose 44 DE mRNA transcripts that were enriched in reproduction-related pathways and all DE lncRNAs to construct a co-expression network. The target genes of all DE lncRNAs were predicted through *cis*- and *trans*-action.

### Verification of the expression pattern of screened DE lncRNAs during testis development

To prove our hypothesis that the screened DE lncRNAs play roles in male reproduction, we examined the expression levels of six DE lncRNAs in sheep testis tissues at prenatal and postnatal ages. The testis tissues of different developmental ages (i.e., prenatal embryo at 110 days and postnatal at 5 days, 3 months, 9 month and 2 years) were collected from 15 Hu sheep (3 sheep per age group), snap frozen in liquid nitrogen, and stored at −80 °C for RNA extraction. qRT-PCR was used for the expression analysis of screened lncRNAs at different developmental ages, and the results were expressed relative to the prenatal embryo at 110 days age group. Finally, the comparative quantification of each RNA was normalized to GAPDH using the 2^−ΔΔCt^ method, and the primers of lncRNAs are shown in Table 4.

### Statistical Analysis

All data were analyzed using the SPSS software (version 20.0). The qPCR data were expressed as the mean ± the standard error of the mean, and the multigroup comparisons of the means were analyzed using one-way analysis of variance (ANOVA) and post hoc contrasts were performed using Tukey’s test. *p* *<* 0.05 was considered statistically significant. Each group contained three samples, and all experiments were repeated at least 3 times.

## Electronic supplementary material

supplemental tables and figures
