# Supplementary material for: Long noncoding RNA expression profile changes associated with dietary energy in the sheep testis during sexual maturation
Source: Sci Rep. 2017 Jul 12;7:5180. doi: 10.1038/s41598-017-05443-5 (PMC5507887; doi:10.1038/s41598-017-05443-5)
Supplement: Supplementary file 1 — supplemental tables and figures [file 41598_2017_5443_MOESM1_ESM.pdf]

Long noncoding RNA expression profile changes associated with dietary energy in the sheep testis during sexual maturation

Yanli Zhang[1], Hua Yang[1], Le Han[1], Fengzhe Li[1], Tingting Zhang[1], Jing Pang[1], Xu Feng[1], Caifang Ren[1], Shengyong Mao[2], Feng Wang[1]

[1]Jiangsu Livestock Embryo Engineering Laboratory, Nanjing Agricultural University, Nanjing 210095, China

[2]Jiangsu Key Laboratory of Gastrointestinal Nutrition and Animal Health, Nanjing Agricultural University, Nanjing 210095, China

Correspondence and requests for materials should be addressed to F.W. (email: caeet@njau.edu.cn)

Table S1. Alignment of statistical results of reads.

Group	Sample	Effective reads	Total mapped	Multiple mapped	Uniquely mapped	Read1 mapped	Read2 mapped	Reads map to '+'	Reads map to '-'
Hay group (Hay)	Hay1	94,146,578(100%)	78,725,626(83.62%)	6,601,523(7.01%)	72,124,103(76.61%)	40,199,831(42.7%)	38,525,795(40.92%)	39,153,438(41.59%)	39,572,188(42.03%)
	Hay2	119,497,828(100%)	98,211,257(82.19%)	7,429,524(6.22%)	90,781,733(75.97%)	50,152,567(41.97%)	48,058,690(40.22%)	48,824,390(40.86%)	49,386,867(41.33%)
	Hay3	91,938,416(100%)	75,501,163(82.12%)	5,122,021(5.57%)	70,379,142(76.55%)	38,569,661(41.95%)	36,931,502(40.17%)	37,533,221(40.82%)	37,967,942(41.3%)
High grain group (HG)	HG1	98,143,376(100%)	83,794,371(85.38%)	4,749,342(4.84%)	79,045,029(80.54%)	43,016,784(43.83%)	40,777,587(41.55%)	41,656,028(42.44%)	42,138,343(42.94%)
	HG2	88,924,920(100%)	70,922,735(79.76%)	4,958,980(5.58%)	65,963,755(74.18%)	36,247,314(40.76%)	34,675,421(38.99%)	35,267,393(39.66%)	35,655,342(40.1%)
	HG3	104,347,880(100%)	87,386,506(83.75%)	7,016,186(6.72%)	80,370,320(77.02%)	44,683,751(42.82%)	42,702,755(40.92%)	43,435,681(41.63%)	43,950,825(42.12%)

1) Effective reads: The number of clean reads.

2) Total mapped: The number of sequences matched to the genome.

3) Multiple mapped: The number of sequences that had multiple positions mapped to the reference sequence.

4) Uniquely mapped: The number of sequences that had unique positions mapped to the reference sequence.

5) Read-1, Read-2 mapped: The number of read-1 and read-2 that locate on the genome; the statistical proportion of the two parts should be substantially the same.

6) Reads map to '+': The number of reads mapped to the positive strand of the genome.

7) Reads map to '-': The number of reads mapped to the negative strand of the genome.

Table S2. DE genes and co-expressed lncRNAs detected in different cell types during sheep spermatogenesis identified by referring to mouse RNA-seq data

Gene	Up-/down regulated	Chromosomal location	Strand	Mouse chromosomal location	Cell-type location	*cis* or *trans* relationship	Co-expressed lncRNAs	Up-/down regulated	Chromosomal location	Strand
IL5RA	Down	Chr19	+	Chr6	priSG-A; plpSC; rST; eST	*trans*	LOC105604050	Up	Chr21	+
						trans	LOC106991431	Down	Chr11	-
OAS2	Up	Chr17	-	Chr5	priSG-A; SG-A; SG-B; plpSC; pacSC; rST; eST	*trans*	LOC105610224	Down	Chr12	+
						trans	LOC105613170	Down	Chr12	+
PROZ	Down	Chr10	+	Chr8	priSG-A; SG-A; SG-B; plpSC; pacSC; rST; eST	*cis*	LOC105607399	Down	Chr10	-
TSHZ2	Down	Chr13	+	Chr2	priSG-A; SG-A; SG-B; plpSC; pacSC; rST; eST	*cis*	LOC105608654	Down	Chr13	-

priSG-A, primitive type A spermatogonia; SG-A, type A spermatogonia; plpSC; SG-B, type B spermatogonia; plpSC, preleptotene spermatocytes; pacSC, pachytene spermatocytes; rST; round spermatids; eST, elongating spermatids.

Table S3. Diet formulas of the two groups.

Item	Diet	
	Hay group	High grain group
Ingredient composition %DM		
Oat hay	66.67	26.67
Alfalfa	33.33	13.33
Corn meal	0	34.2
Wheat meal	0	18
Soybean	0	4.2
CaCO3	0	1.08
NaCl, salt	0	0.42
CaHPO4	0	1.2
Mineral and vitamin supplement	0	0.9
Nutrient composition		
Metabolic energy, MJ/kg DM	7.24	14.79
Crude protein, % DM	12.30	12.04
Crude fat, % DM	3.39	3.45
Crude fiber, % DM	28.94	12.51
Neutral detergent fiber, % DM	53.60	27.38
Acid detergent fiber, % DM	33.41	15.57
Crude ash, % DM	7.87	6.71
Starch, % DM	2.03	31.58
Ca, % DM	0.79	1.02
P, % DM	0.30	0.49

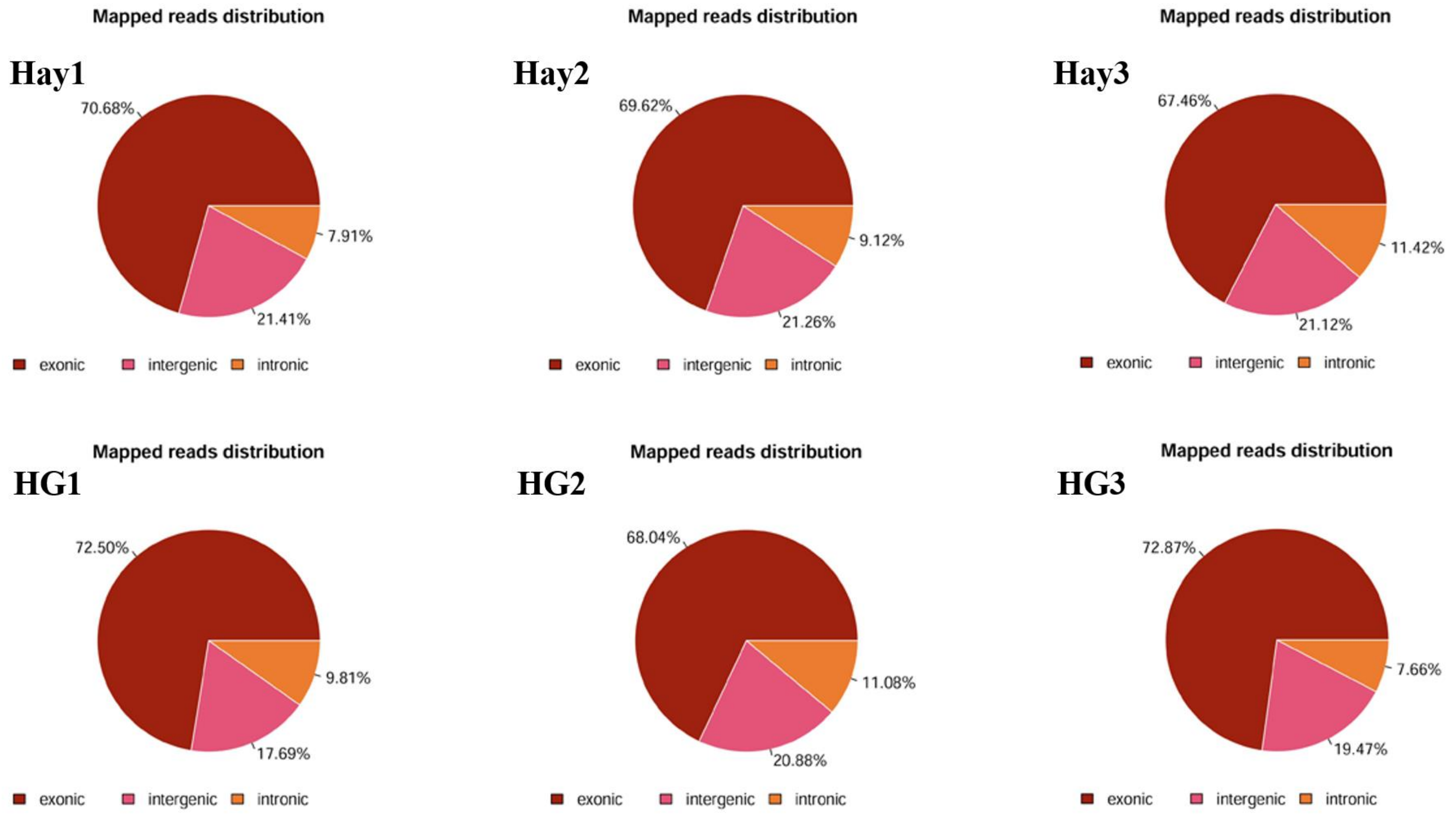

Fig. S1. Distribution of reads mapped to the genome. The brown, pink, and orange colors represent the exonic, intergenic, and intronic regions, respectively.

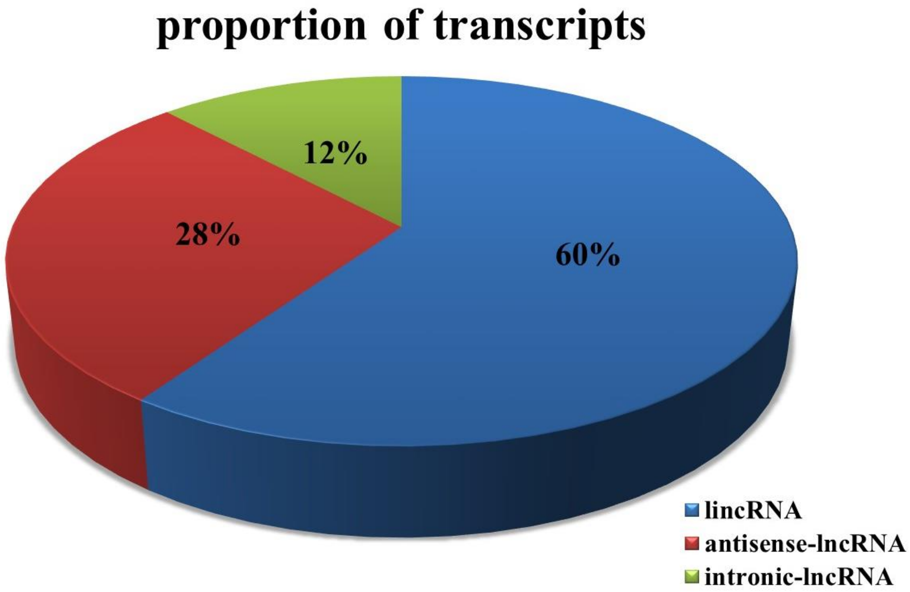

Fig. S2. Classification and proportion of lncRNA transcripts. The blue, red, and green colors represent lincRNA, antisense-lncRNA, and intronic-lncRNA, respectively.

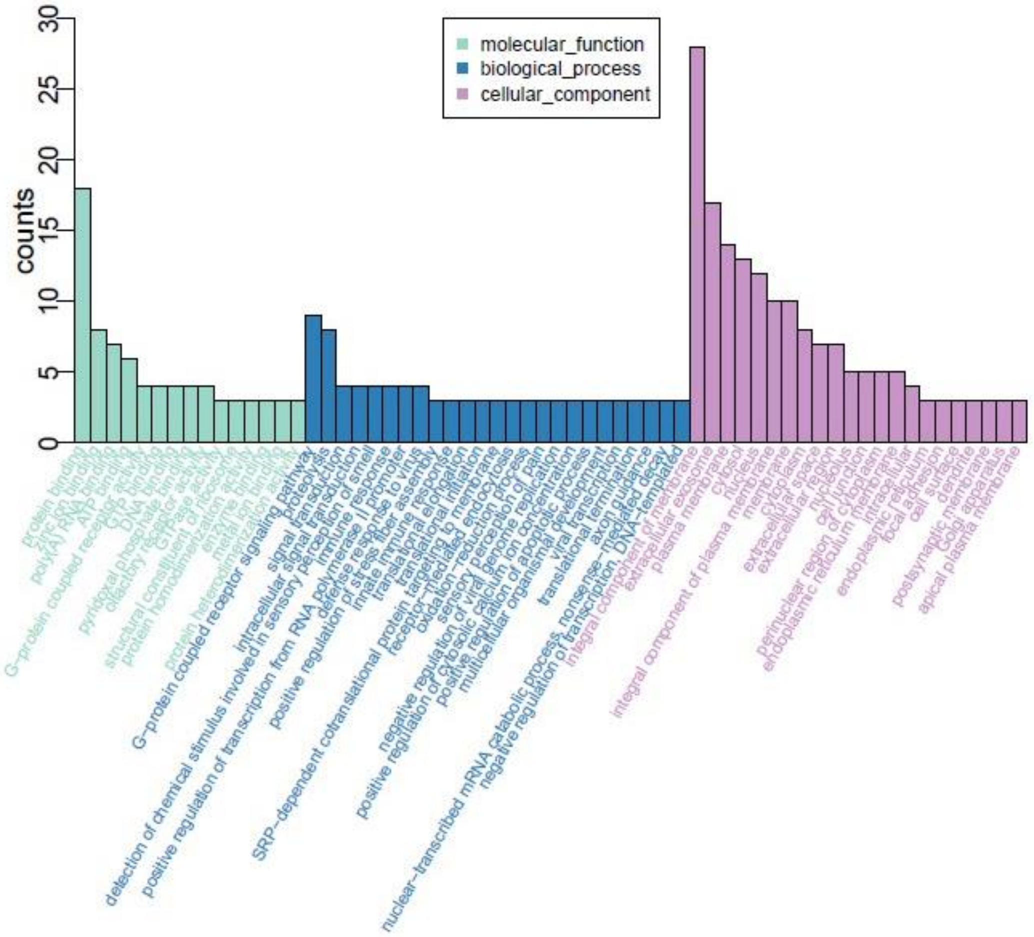

Fig. S3. GO enrichment analysis of DE genes. The light-blue, dark-blue, and purple colors indicate molecular functions, biological processes, and cellular components, respectively.

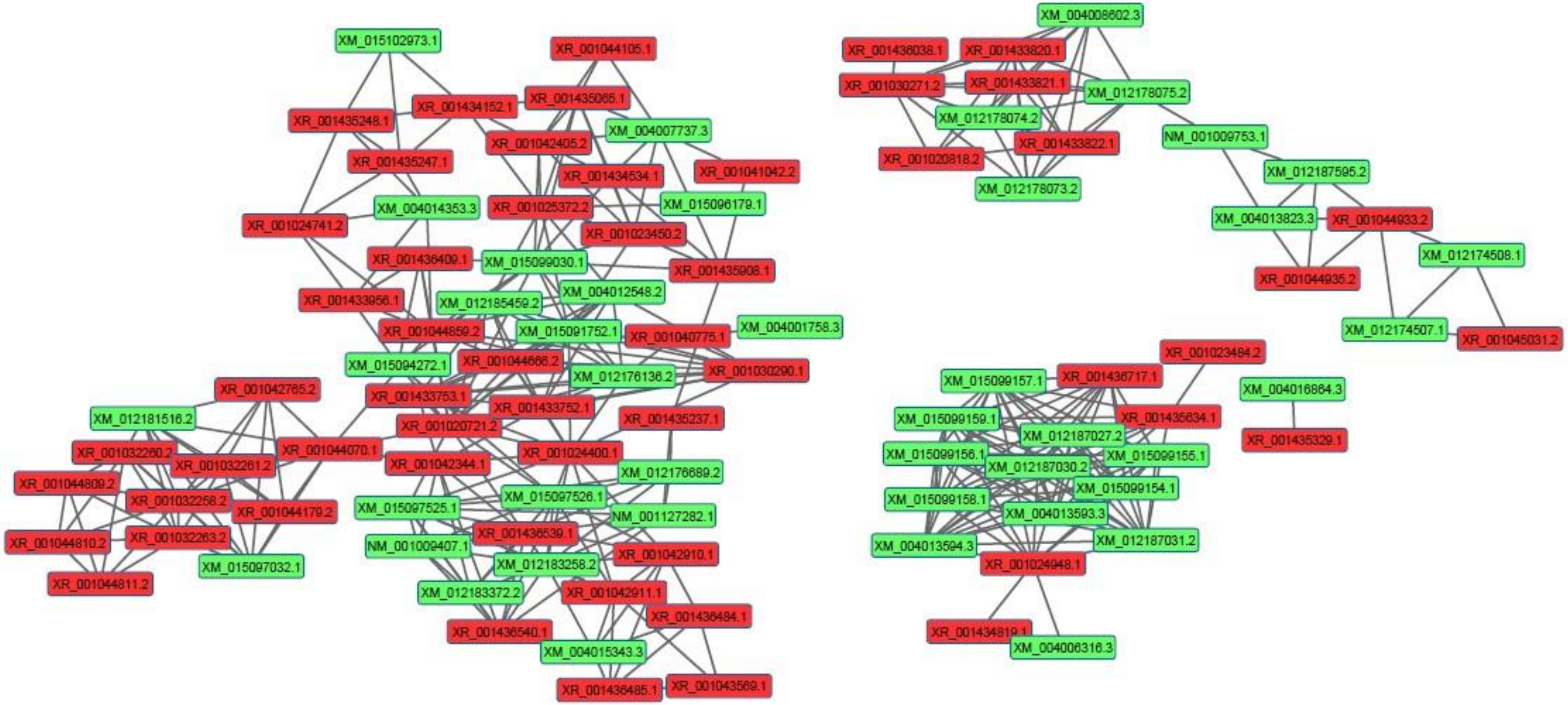

Fig. S4. Network between 43 DE lncRNAs and 22 DE mRNAs. mRNAs are shown in green and lncRNAs are shown in red.

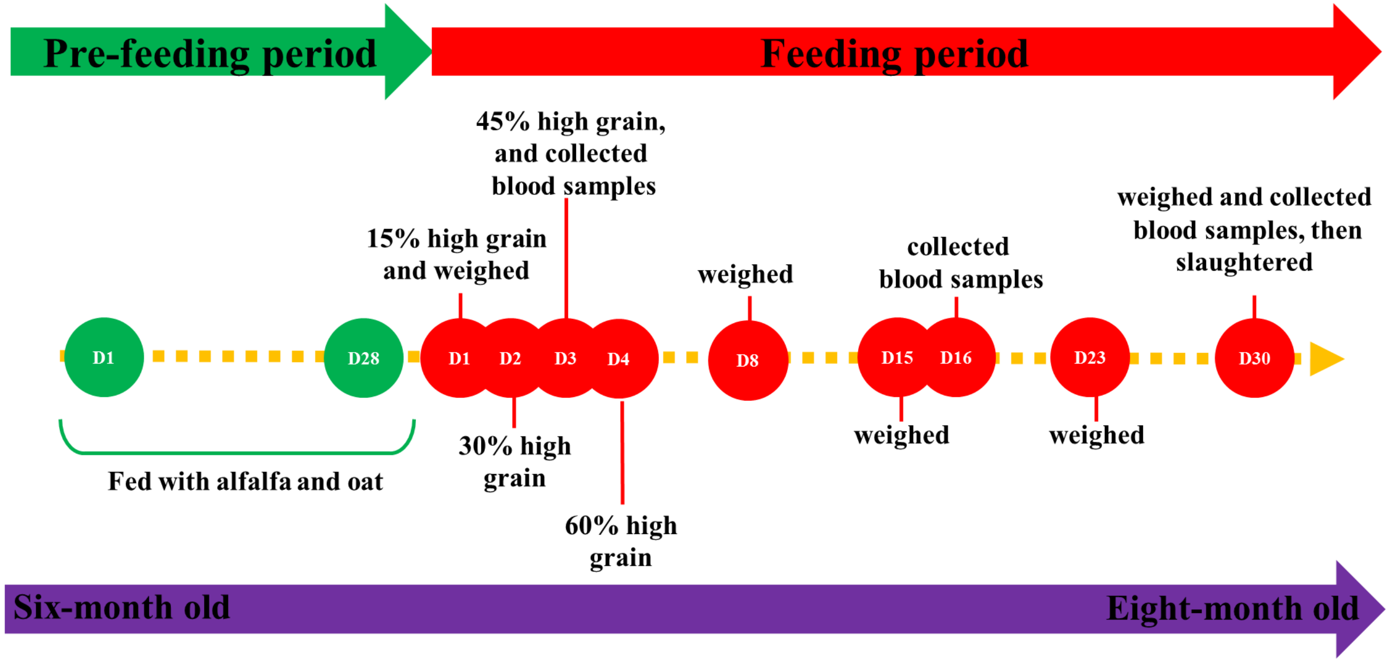

Fig. S5. The feeding scheme of the experiment. The diagram shows the feeding scheme used for the high grain group. The feeding scheme used for the hay group was similar to that used for the high grain group, except that the hay group was only fed alfalfa and oats during the experimental period.

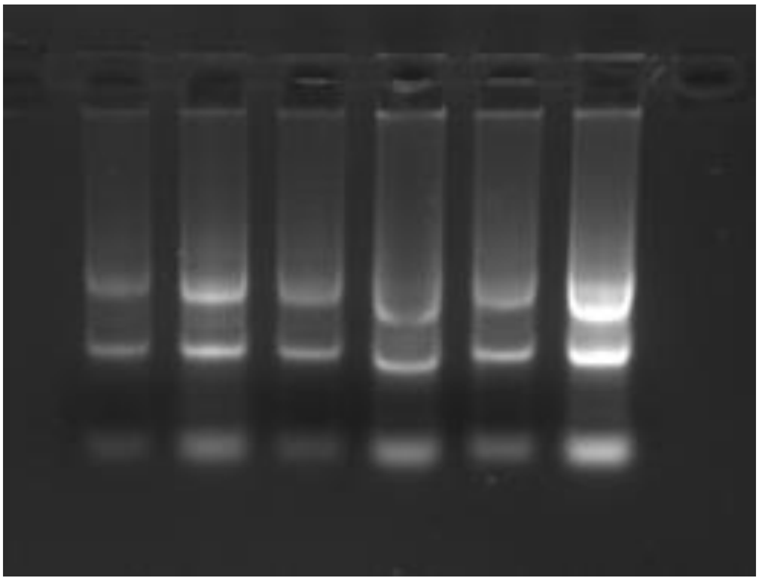

Fig. S6. Agarose gel electrophoresis analysis of RNA-extracted products of sheep testis.
The agarose gel electrophoresis was visualized under UV illumination with 50ms exposure time.

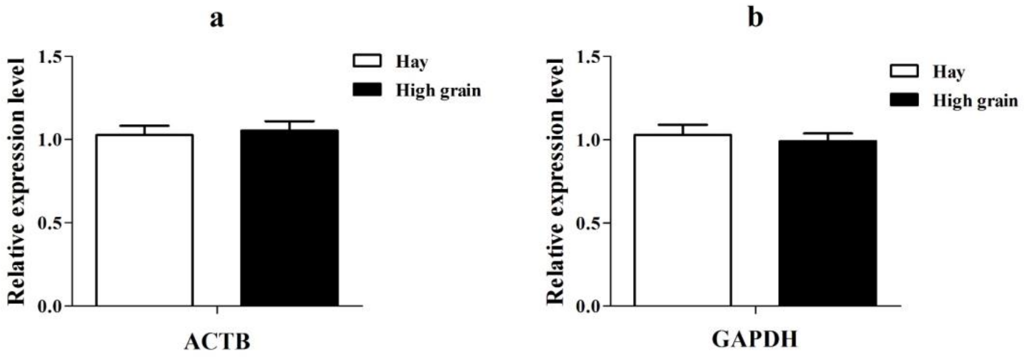

Fig. S7. Expression level of *ACTB* normalized to *GAPDH* (a) and expression level of *GAPDH* normalized to *ACTB* (b).

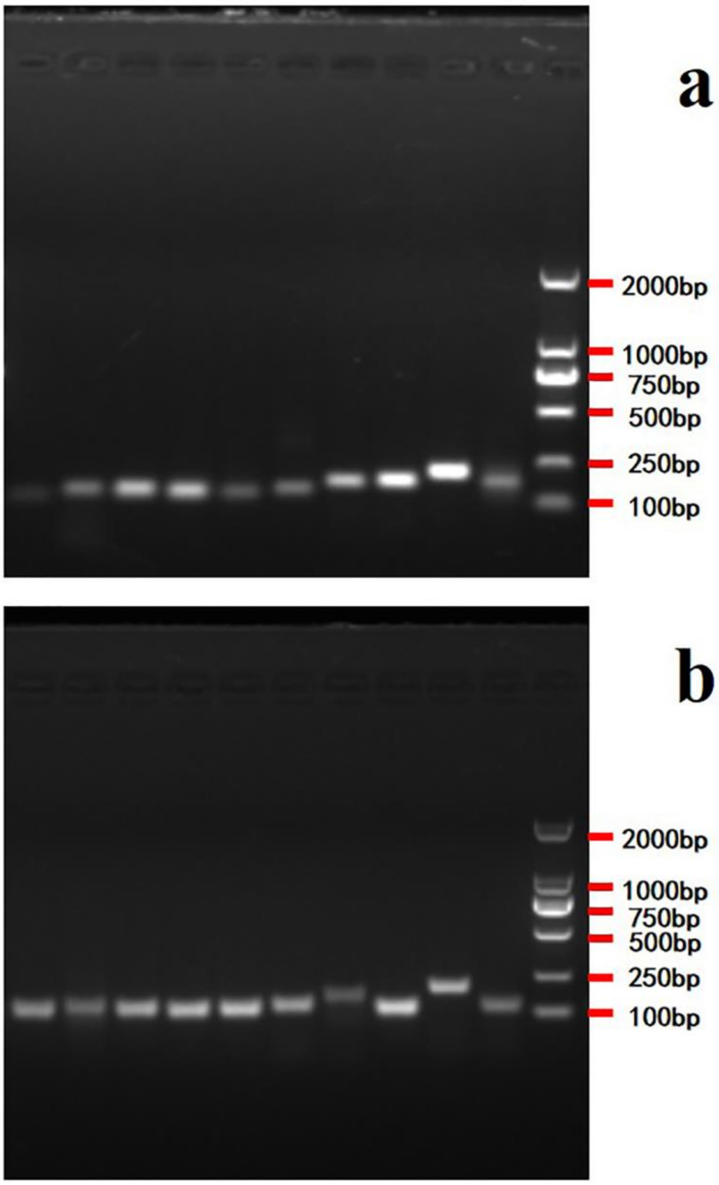

Fig. S8. Agarose gel electrophoresis analysis of the primers of differentially expressed mRNAs and lncRNAs are shown in a and b, respectively. The differentially expressed mRNAs and lncRNAs were chosen randomly from the sequencing data and the agarose gel electrophoresis was visualized under UV illumination with 50ms exposure time. The bands in part a represent XM_004008021.3, XM_012173604.2, XM_004018909.3, XM_004006316.3, XM_004012176.3, XM_012184864.2, XM_004008307.3, XM_015101162.1, XM_015097032.1, and XM_012104750.2 from left to right; in part b, the bands represent the differentially expressed lncRNAs: XR_001045031.2, XR_001040775.1, XR_001434819.1, XR_001044933.2, XR_001044935.2, XR_001044859.2, XR_001435329.1, XR_001025228.2, XR_001043569.1, and XR_001042765.2 from left to right.
